# Moving HER2-low breast cancer predictive and prognostic data from clinical trials into the real world

**DOI:** 10.3389/fmolb.2022.996434

**Published:** 2022-09-26

**Authors:** Serena Di Cosimo, Eliana La Rocca, Silva Ljevar, Maria Carmen De Santis, Marta Bini, Vera Cappelletti, Marta Valenti, Paolo Baili, Filippo G. de Braud, Secondo Folli, Gianfranco Scaperrotta, Chiara Volpi, Andrea Vingiani, Claudio Vernieri, Paolo Verderio, Rosalba Miceli, Giancarlo Pruneri

**Affiliations:** ^1^ Integrated Biology Platform Unit, Department of Applied Research and Technological Development, Fondazione IRCCS Istituto Nazionale dei Tumori, Milan, Italy; ^2^ Radiation Oncology 1, Fondazione IRCCS Istituto Nazionale dei Tumori, Milan, Italy; ^3^ Breast Unit, Fondazione IRCCS Istituto Nazionale dei Tumori, Milano, Italy; ^4^ Clinical Epidemiology and Trial Organization Unit, Department of Applied Research and Technological Development, Fondazione IRCCS Istituto Nazionale dei Tumori, Milan, Italy; ^5^ Biomarkers Unit, Department of Applied Research and Technological Development, Fondazione IRCCS Istituto Nazionale dei Tumori, Milan, Italy; ^6^ Analytic Epidemiology and Health Impact Unit, Fondazione IRCCS Istituto Nazionale dei Tumori, Milan, Italy; ^7^ Department of Medical Oncology, Fondazione IRCCS Istituto Nazionale dei Tumori, Milano, Italy; ^8^ School of Medicine, University of Milan, Milan, Italy; ^9^ Radiology Unit, Fondazione IRCCS Istituto Nazionale Tumori, Milano, Italy; ^10^ Department of Pathology, Fondazione IRCCS Istituto Nazionale Dei Tumori, Milano, Italy; ^11^ IFOM ETS - the AIRC Institute of Molecular Oncology, Milan, Italy; ^12^ Bioinformatics and Biostatistics Unit, Fondazione IRCCS Istituto Nazionale Tumori, Milano, Italy

**Keywords:** HER2-low, breast cancer, neoadjuvant chemotherapy, predictive, prognostic

## Abstract

**Background:** Previous data, mostly from clinical trials, reported that HER2-low status is associated with low pathological complete response (pCR), and favourable prognosis. Since these findings suggest the existence of an additional breast cancer subtype, we questioned if the predictive/prognostic value of HER2-low was also relevant in the real world.

**Methods:** Data from non-metastatic breast cancer patients treated with neoadjuvant chemotherapy and surgery (2009–2020) were retrieved from our institutional prospectively-maintained registry. Univariable and multivariable logistic models were implemented to study the association between pCR and baseline HER2 status. Univariable analysis of disease-free survival (DFS) was performed through Kaplan-Meier survival curves and log-rank tests.

**Results:** Starting from a total of 790 consecutive cases, we identified 444 newly-diagnosed breast cancer patients featuring HER2 immunohistochemistry (IHC) 0 (HER2-0, *n* = 109), and 1 + or IHC 2+/*in situ* hybridization negative (HER2-low, *n* = 335) receiving anthracycline and taxane-based regimens in 88.9% of cases. Most of the patients were diagnosed with stage II (67.3%) and there was no difference of disease presentation according to HER2-status. pCR was attained by 71 (16.0%) patients and was significantly associated with increased DFS (*p* = 0.031). Compared to HER2-0, HER2-low cases were more likely hormone receptor-positive (81.2% vs. 43.1%, *p* < 0.001), well-differentiated (47.5% vs. 26.6%, *p* = 0.001), less proliferative (21.5% vs. 8.3%, *p* = 0.001) and less responsive to treatment (pCR 11.6% vs. 29.4%, *p* < 0.0001). There was no difference in DFS according to HER2 status, though hormone-receptor (HR) negative/HER2-low cases tended to have a worse prognosis compared to HR-negative/HER2-0. By pCR achievement, 3-years DFS was 87.5.% (75.1–100%) vs. 71.6% (65.9–77.8%) (*p* = 0.161) in HER2-low and 89.1% (75.8–100%) vs. 72.1% (59.7–87.0%) (*p* = 0.092) in HER2-0.

**Conclusion:** Our real-world data show that HER2-low breast cancer patients represent roughly a half of the cases treated with neoadjuvant therapy, and have poor treatment response. In absence of pCR, HER2-low breast cancer patients have a dismal prognosis, especially when primary tumor hormone receptor status is negative. Studies are therefore needed to define the biology of these tumors for new therapeutic targets and to incorporate HER2-targeting agents in early-stage treatment.

## Introduction

Human epidermal growth factor receptor 2 (HER2) is a member of the epidermal growth factor receptor (EGFR) family, which is composed by EGFR/HER1, HER2, HER3, and HER4 (Yarden and Sliwkowski, 2001). The HER2 extracellular domain has no known ligand and is activated by the formation of homo or heterodimers (Tzahar et al., 1996). These dimers lead to phosphorylation of tyrosine kinase residues in the cytoplasmic domain which function as docking sites for proteins that in turn activate the phosphatidylinositol triphosphate kinase (PI3K) and mitogen-activated protein kinase (MAPK) signaling pathways, leading to cell cycle progression and proliferation (Citri and Yarden, 2006). Breast cancer cases with HER2 amplification and/or overexpression show up to 25–50 copies of the HER2 gene, and 40–100-fold increase in HER2 protein resulting in two million receptors expressed at the cell surface (Slamon et al., 1989). HER2 overexpression and amplification are routinely tested by immunohistochemistry (IHC) and *in situ* amplification (ISH) to identify patients with IHC 3 + or 2+/ISH+ (HER2-positive) who may benefit from HER2-targeted therapy (e.g., trastuzumab and pertuzumab), which suppresses HER2-driven intracellular signaling and markedly improves survival (Baselga et al., 2017). More recently, the possibility of targeting HER2 has been extended to patients with breast cancer featuring HER2 IHC 1 + or IHC2+/ISH-, so called breast cancer cases with low HER2 expression (HER2-low). Specifically, the new class of anti-HER2 agents represented by antibody drug conjugates (ADCs) have shown to bind HER2, to enter the cell, and to leave their membrane-permeable topoisomerase I inhibitor payload causing a catastrophic DNA damage in both the targeted and neighboring tumor cells (Ponde et al., 2019). The bystander antitumor effect, offered by the optimized ADC technology of trastuzumab-deruxtecan (T-DXd), has been proposed to exert its activity in patients whose tumors have varying HER2 expression including HER2-low (Doi et al., 2017; Modi et al., 2020). Recently, the DESTINY-Breast04 study demonstrated the superior anti-tumor activity of T-DXd over physicians’choice in advanced breast cancer patients with HER2-low primary tumors (Modi et al., 2022). These results provided motivation to understand both the distribution and prognostic significance of HER2-low status in breast cancer. If, for example, HER2-low status were associated with a poorer prognosis than HER2-0 status, this would prompt consideration of the usefulness of anti-HER2 therapies in HER2-low patients at a very early stage of the disease (Pernas and Tolaney, 2020). These results provided motivation to understand both the clinico-pathological characteristics and prognostic relevance of HER2-low status in breast cancer. In this regard, in a pooled analysis of four prospective neoadjuvant clinical trials, Denkert et al. (Denkert et al., 2021) provided evidence that HER2-low status was associated with low pathological complete response (pCR), and favourable clinical outcome. Since this initial publication, many authors have proposed that HER2-low breast cancer may represent an additional and distinct breast cancer subtype (Tarantino et al., 2020). However, most of these analyses were performed in the context of clinical trials, with controlled sample population, whose findings could be potentially limited by the characteristics of the cohort evaluated in the trial. Herein, we questioned if the predictive/prognostic value of HER2-low status was also relevant in the real world. For this purpose, we tested the hypothesis that patients with breast cancer featuring low levels of HER2 had different clinical-pathological characteristics and survival outcome compared with HER2-0 in breast cancer patients routinely treated with neoadjuvant chemotherapy in the largest public comprehensive cancer center of Italy.

## Methods

### Study population

Newly diagnosed breast cancer cases receiving neoadjuvant chemotherapy between May, 2009 and December, 2020 were identified in a prospectively maintained pathology-based registry at Fondazione IRCCS Istituto Nazionale dei Tumori-Milano, Italy (Baili et al., 2015). Pathological tumor stage was assigned according to the seventh TNM edition (American Joint Committee on Cancer, 2010). Hormone receptor status was classified as positive when either Estrogen (ER) or Progesterone receptor (PR) was ≥ 1%, or negative when both ER and PR receptors were < 1% (Hammond et al., 2010). HER2 status was considered positive when (a) the immunohistochemistry (IHC) score was 3 + or (b) the IHC was 2 + and chromogenic *in situ* hybridization was diagnostic of gene amplification (ISH+). HER2 was zero when the IHC score was 0, and HER2-low when IHC was 1 + or 2 +/ISH-. Ki67 was evaluated by IHC. Specifically, the assessment was performed in a selected representative block of each tumor by selecting at least three fields of “hot spots” at the periphery of tumor edge of invasion and counting ratio between stained and unstained nuclei of about 500 cells. pCR was defined as no residual invasive cancer was found in breast and lymph nodes at surgery.

### Statistical analysis

Patient and disease characteristics were summarized by descriptive statistics.

The Kruskal–Wallis or the Mann-Whitney-Wilcoxon test, as appropriate, was used to analyze the association between HER2 status or pCR and numerical variables, whereas the Fisher-Freeman-Halton test was used when analyzing association with categorical variables (Freeman and Halton, 1951); patients with missing data were excluded. The association between pCR and HER2 status was studied using univariable and multivariable logistic regression models, the latter by adjusting for those variables that were statistically significant in univariate analysis, specifically patient menopausal status (derived by patient age, i.e., < 50 years premenopausal; ≥ 50 years postmenopausal), clinical tumor size, grading, hormone receptor status and Ki67. The model results are reported in terms of pCR odds ratios (OR) and corresponding 95% confidence intervals (95% CIs); ORs > 1 indicate a greater chance of pCR associated to a covariate category (or value) versus (vs.) the reference one, and CIs not including the value of one are suggestive of significant association between pCR and the covariate.

DFS was defined as the time from the date of surgery to the date of loco-regional or distant recurrence or death from any cause, whichever occurred first. DFS curves were estimated with the Kaplan–Meier method and between groups differences were tested using the log-rank test.

All statistical analyses were performed using the R software {R Development Core Team (2007). R: A language and environment for statistical computing. R Foundation for Statistical Computing, Vienna, Austria. ISBN 3–900,051–07–0, http://www.R project.org [accessed 30 May 2021]} and SAS software (Version 9.4; SAS Institute, Inc., Cary, NC).

## Results

**FIGURE 1:**
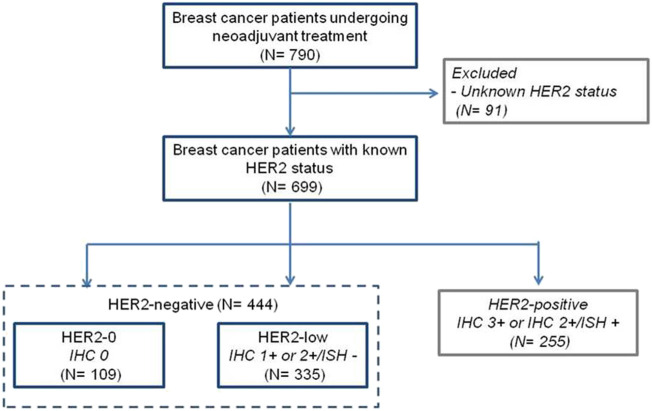
CONSORT.

Data were obtained from 790 newly-diagnosed breast cancer patients undergoing neoadjuvant therapy, including 699 with known HER2-status. For the purpose of this study, we analyzed 444 women with baseline HER2-negative breast cancer based on IHC and ISH results (Figure 1). Specifically, we considered 335 (75.5%) cases with primary tumor biopsy IHC 1+ and 2+/ISH- as HER2-low, and 109 (24.5%) with IHC 0 as HER2-0. Baseline patient and primary tumor characteristics are shown in Table 1. Compared to HER2-0, HER2-low cases were most often HR-positive (81.2% vs. 43.1%, *p* < 0.001), and presented with more differentiated (47.5% vs. 26.6%, *p* = 0.001), and less proliferative primary tumors (21.5% vs. 8.3%, *p* = 0.001) (Table 1). An additional analysis by HR status showed that HER2-low breast cancer occurred more frequently in younger and pre-menopausal patients among HR-positive cases; and was associated with worse histological grade among HR-negative cases (Supplementary Tables S1,S2).

**TABLE 1:** Baseline patient and primary tumor characteristics according to HER2-0 and HER2-low status.

	HER2-0 (*n* = 109)	HER2-low (*n* = 335)	*p*-value^*^
Age			0.3742
Mean (SD)	51.9 (11.0)	50.8 (11.6)	
Median [Q1, Q3]	51.2 [43.9, 59.1]	49.0 [43.1, 59.0]	
Menopausal status			0.1860
Yes	51 (46.8%)	182 (54.3%)	
No	58 (53.2%)	153 (45.7%)	
BMI			0.8656
Mean (SD)	24.9 (5.05)	24.8 (4.72)	
Median [Q1, Q3]	24.1 [21.2, 27.5]	24.1 [21.3, 27.7]	
Missing	9 (8.3%)	34 (10.1%)	
Neoadjuvant chemotherapy			0.2949
Anthracycline and taxane	94 (86.2%)	301 (89.9%)	
Other	15 (13.8%)	34 (10.1%)	
Clinical tumor size			0.5660
2–5 cm	78 (71.6%)	233 (9.6%)	
> 5 cm	29 (26.6%)	99 (29.6%)	
Missing	2 (1.8%)	3 (0.9%)	
Clinical nodal status			0.5284
N0	36 (33.0%)	95 (28.4%)	
N1	65 (59.6%)	215 (64.2%)	
N2- 3	7 (6.4%)	17 (5.1%)	
missing	1 (0.9%)	8 (2.4%)	
Grading			0.0010
I	1 (0.9%)	14 (4.2%)	
II	28 (25.7%)	145 (43.3%)	
III	61 (56.0%)	140 (41.8%)	
missing	19 (17.4%)	36 (10.7%)	
Estrogen and Progesterone receptor status			<0.0001
Both negative	62 (56.9%)	63 (18.8%)	
At least one positive	11 (10.1%)	45 (13.4%)	
Both positive	36 (33.0%)	227 (67.8%)	
Ki67			0.0041
< 20%	9 (8.3%)	72 (21.5%)	
≥ 20%	95 (87.2%)	246 (73.4%)	
missing	5 (4.6%)	17 (5.1%)	
Surgery			0.0149
Mastectomy	60 (55.0%)	229 (68.4%)	
BCS	49 (45.0%)	106 (31.6%)	
Axillary dissection			0.0002
Yes	43 (39.4%)	201 (60.0%)	
No	66 (60.6%)	134 (40.0%)	
Pathological complete response			<0.0001
Yes	32 (29.4%)	39 (11.6%)	
No	77 (70.6%)	296 (88.4%)	
Number and type of events^^^			
Loco-regional relapse (LRR)	3	15	
Metastases	7	52	
LRR and metastases	2	15	

* The associations were tested using Wilcoxon-Mann-Whitney test for continuous variables and Fisher’s exact test for categorical variables. Missing values were excluded from statistical tests.

^ Reported over 75 and 287 HER2-0 and HER2-low, respectively with a median follow-up of 59.6 (Interquartile Range [IQR] 37.0–88.4) months.

### Association between HER2-low and pathological complete response

**FIGURE 2:**
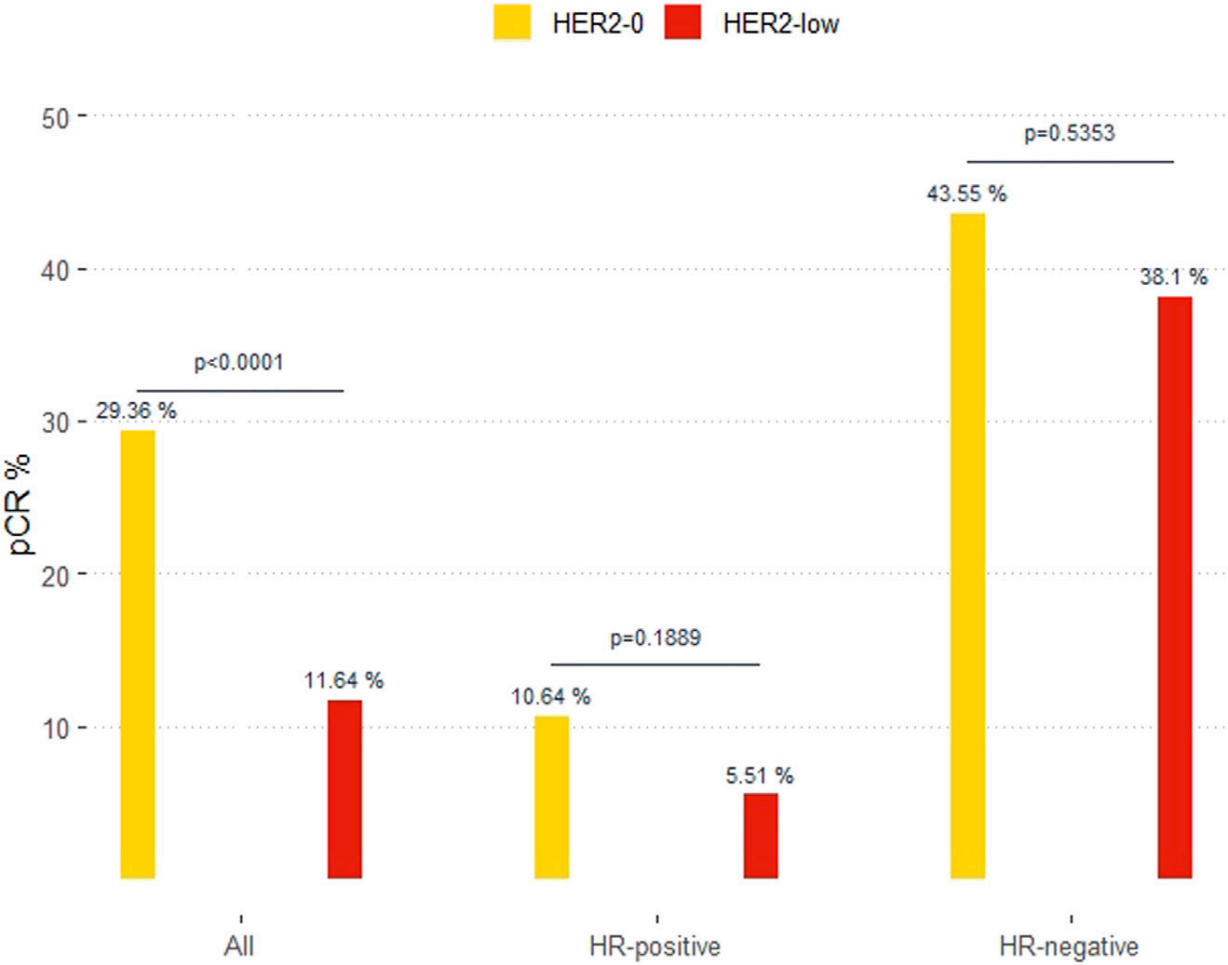
Pathological complete response (pCR) in HER2-0 and HER2-low overall and by hormone-receptor (HR) status.

In the overall cohort, 16% (71 of 444) of patients achieved a pCR after receiving mostly (88.9%) anthracycline and taxane-based chemotherapy. Patients with primary tumors featuring low HER2 expression had significantly lower pCR than those with HER2-0, 11.6% vs. 29.4%, OR 0.32 (95%CI 0.19–0.54; *p* < 0.0001). According to HR status, pCR were 10.6% and 5.5% (*p* = 0.18) in HER2-0 and HER2-low cases with HR-positive; and 43.6% and 38.1% (*p* = 0.53) in HER2-0 and HER2-low cases with HR-negative (Figure 2).

In the bivariable model with HR-status, HER2-low cases showed a lower OR for pCR in HR-positive (0.49, 95%CI 0.17–1.42) than in HR-negative cases (0.80, 95%CI 0.39–1.63), *p*-value for interaction HER2 x HR = 0.4564 (Table 2). After adjusting for menopausal status, grade, tumor size and Ki67, OR was 0.52 (95%CI 0.17–1.54) in HR-positive and 0.64 (95%CI 0.30–1.35) in HR-negative cases.

**TABLE 2:** Association between clinical-pathological variables and pathological complete response in the overall study population.

Univariable logistic models				
Variable	OR	Lower 0.95	Upper 0.95	*p*-value
HER2				<0.0001
HER2-low vs. HER2-0	0.32	0.19	0.54	
Age	0.75	0.52	1.08	0.1169
Menopausal status				0.0463
Yes vs. No	0.59	0.35	0.99	
BMI	1.24	0.89	1.74	0.2006
Chemotherapy				0.0128
Anthracycline and taxane-based vs. other	0.42	0.21	0.83	
Stage				0.2354
III vs. I/II	0.69	0.38	1.27	
Grade				<0.0001
I-II vs. III	0.14	0.07	0.29	
missing vs. III	0.46	0.21	1.04	
HR-status				<0.0001
Positive vs. Negative	0.10	0.05	0.17	
Ki67				0.0065
Low vs. High	0.10	0.02	0.42	
missing vs. High	0.00	0.00	>10000	
Bivariable logistic model				
HER2-status				0.3481
*In HR-negative*	0.80	0.39	1.63	
HER2-low vs. HER2-0				
*In HR-positive*	0.49	0.17	1.42	
HER2-low vs. HER2-0				
HR status				<0.0001
Positive vs. Negative	0.15	0.05	0.44	
Interaction HER2 status × HR status				0.4564
Multivariable logistic model				
Variable	OR	Lower 0.95	Upper 0.95	*p*-value
HER2-status				0.2518
*In HR-negative*	0.64	0.30	1.35	
HER2-low vs. HER2-zero				
*In HR-positive*	0.52	0.17	1.54	
HER2-low vs. HER2-zero				
Menopausal status				0.0651
Yes vs. No	0.57	0.32	1.04	
Clinical tumor size				0.6864
> 5 cm vs. ≤ 5 cm	0.87	0.44	1.71	
Grade				0.2049
I-II vs. III	0.45	0.19	1.08	
missing vs. III	0.78	0.31	1.97	
HR-status				<0.0001
Positive vs. Negative	0.22	0.07	0.66	
Ki67				0.2638
Low vs.High	0.29	0.06	1.32	
Low vs. Missing	0.00	0.00	>10000	
Interaction HER2 status × HR status				0.7494

Pre-menopausal status (*p* = 0.046), grade III (*p* < 0.001), Ki67 > 20% (*p* = 0.007) and lack of HR expression (*p* < 0.001) were also shown to predict pCR in univariable analysis. Of all these variables, only HR-status retained its predictive value for response to neoadjuvant chemotherapy in multivariable analysis (OR = 0.19, 95%CI 0.10–0.37; *p* = 0.0001; Table 2).

### Association between HER2-low and disease free survival

**FIGURE 3:**
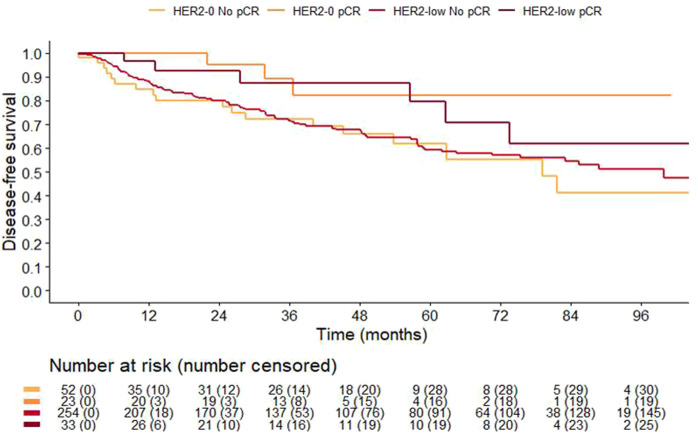
Disease-free Survival according to pCR and HER2-status.

Overall, 82% of patients (362 of 444) were monitored at our Institute for a median follow-up time of 59.6 (Interquartile Range [IQR] 37.0–88.4) months. As expected, patients achieving a pCR had a significantly improved 3-years DFS compared to those with residual disease 88% (95%CI 78–99%) vs. 72% (95%CI 66–77%) (*p* = 0.031). Noteworthy pCR retained its favorable prognostic value independently of HER2-status. Specifically, 3-years DFS of HER2-low patients with or without a pCR was 88% (95%CI 75–100%) and 72% (95%CI 66–78%) (*p* = 0.16), respectively. Similarly, 3-years DFS among HER2-0 patients with and without a pCR was 89% (95%CI 76–100%) and 72% (95%CI 60–87%, *p* = 0.092), respectively (Figure 3). There was no difference in clinical outcome according to HER2 status overall (3-years DFS was 78% (68–89%) and 73% (68–79%) in HER2-0 and HER2-low, *p* = 0.8533), and according to HR-status. However, HR-negative cases with HER2-low had a poorer 3-years DFS compared to HER2-0, 55% (95%CI 42–72%) vs. 73% (95%CI 61–89%), respectively (*p* = 0.097; Supplementary Figure S1A), while no difference was observed in HR positive cases (Supplementary Figure S1B). This trend towards poorer prognosis was especially evident among patients not achieving a pCR (Supplementary Figure S2).

### HER2 status before and after neoadjuvant therapy

**FIGURE 4:**
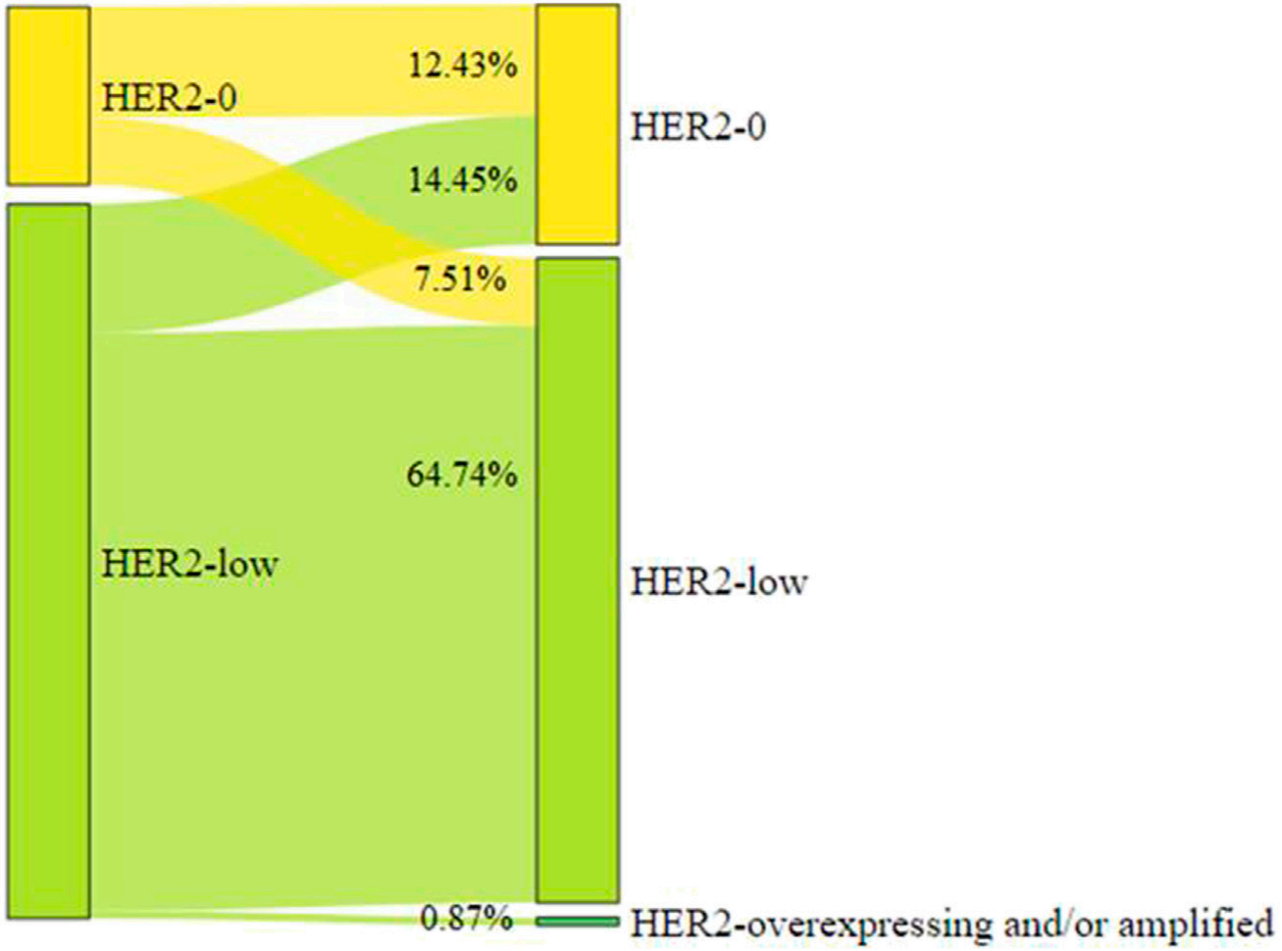
Evolution of HER2 status before and after neoadjuvant chemotherapy.

Paired tumor tissue samples of diagnostic biopsy and surgical samples were available in 346 of 444 cases (77.9%). Fifty of 346 (14.5%, 95%CI 10.9–18.6) patients showed a change from HER2-low to HER2-0. Twenty-six of 346 (7.51%, 95%CI 4.97–10.82) patients converted from HER2-0 to HER2-low (Figure 4).

### HER2 status at relapse

Matched primary tumors and/or surgical samples and recurrent disease were available for 28 patients over the total of 94 who eventually relapsed (11 of 18 loco-regional recurrences (LRR), 11 of 59 metastatic disease, and six of 17 both LRR and metastatic disease). Overall, 23 of 28 recurrent cases (82.1%) were HER2-low. HER2-status at relapse overlapped that of end-of-treatment residual disease in 20 (71.4%) cases. Discordance in HER2 status occurred in 8 (28.5%) cases as following: from residual HER2-0 to relapsed HER2-low (*n* = 5), and *viceversa* (*n* = 3).

## Discussion

To the best of our knowledge, this is the largest attempt to use real-world data to investigate differences in clinical-pathological characteristics and oncological outcome in HER2-negative breast cancer patients treated with neoadjuvant therapy. Several key findings with clinical relevance were identified.

First, patients with breast cancer featuring a low HER2 expression represent 48% of cases treated in the neoadjuvant setting with primary anthracycline and taxane-based chemotherapy in current clinical practice. Previous studies analyzed the distribution of HER2-low breast cancer within the HER2-negative population, reporting results ranging from 47.5% to 59.7% (Shui et al., 2020; Denkert et al., 2021; Schettini et al., 2021). However, those studies were criticized for their heterogeneity, as they used a combination of multiple databases (Denkert et al., 2021), with different inclusion/exclusion criteria (Schettini et al., 2021), and lacked standardized assessments in the different enrolment centers (Shui et al., 2020). Hence, our results complement previous work because they refer to a homogeneous population of women mostly diagnosed with stage II disease, treated with standard care and followed-up in a single centre, which prevented heterogeneity in patient selection, type of treatment and HER2 assessment of multicenter laboratories.

Secondly, we report that 68% of HER2-low tumors are also HR-positive, which is consistent with prior results in the literature ranging from 64% to 88% (Denkert et al., 2021; Schettini et al., 2021). Given the role of HER2 in the pathogenesis of breast cancer, we would expect that its expression even when low would confer more aggressive features than its total absence. By contrast HER2-low breast cancer presented more akin to luminal-like in the majority of cases. One likely explanation for this paradox is that HER2 signaling is modulated by the presence of other HER family members, such as HER3 which is associated with favorable clinical features and a prognostic advantage in HER2-positive tumors (reviewed in 1). Furthermore, the cross-talk between HER2 and ER pathways results in favorable clinical presentation and prognosis and reduced response to anti-HER2 treatment in HER2-positive breast cancer (Yarden and Sliwkowski, 2001; Baselga et al., 2017). That said, literature data exploiting the PAM50 gene expression classifier suggest that differences in HR expression between HER2-low and HER2-0 may reflect differences in molecular subtype distribution. Specifically, HER2-low breast cancer were classified as luminal A, B, basal-like, and HER2-enriched in 50.8%, 28.8%, 13.4%, and 3.5% of cases; whereas 1,486 HER-0 in 28.7%, 18.9%, 43.7%, and 5.9% of cases, respectively (Schettini et al., 2021). Consistently, HER2-low tumors showed a higher expression of luminal-related genes (e.g., *BCL2, BAG1, FOXA1, ESR1*), and a lower expression of basal-like and proliferation-related genes in comparison to HER2-0 (Schettini et al., 2021). Such transcriptomic differences between HER2-low and 0 were independently confirmed on an independent series from the TCGA dataset (Agostinetto et al., 2021). Moreover also at genomic level, HER2-low tumors displayed a different somatic mutation landscape and mutated pathways, with higher *PIK3CA* and lower *TP53* mutations than HER2-0 tumors (Denkert et al., 2021; Zhang et al., 2022). Taken together these evidence definitely support the higher prevalence of luminal disease among HER2-low cases. Importantly, our clinic-pathological findings, while in the real-world setting, mirror such unique biological background, as HER2-low cases were less aggressive, and slowly proliferative. From a therapeutic point of view, the characteristics of HER2-low tumors make them unresponsive to treatment. Indeed, we reported a pCR as low as 11% in HER2-low cases, which fell to 5.5% in HR-positive cases, confirming the prior results of Denkert et al. from data obtained from clinical trials (Denkert et al., 2021).

Thirdly, our study provides information on the prognosis of HER2-low and HER-0 breast cancer patients overall and according to response to treatment. Survival data from already published studies are far from consistent. Ignatov et al. reported that patients with intermediate score (IHC 2 + and ISH-) had a worse prognosis than patients with IHC 0 or 1 + (Ignatov et al., 2015). Schettini et al. (17) found no differences between HER2-low and HER2-0 groups, and Denkert et al. (11) observed an improved 3-years DFS in HER2-low than that HER2-0 patients. Herein, we found no difference in DFS according to HER2 status. Nevertheless, while patients with HR-positive had a similar DFS regardless the expression of HER2, those with HR-negative has a worse outcome when HER2 was low instead of zero (Supplementary Figure S1). Our finding is in line with the recently reported analysis of triple negative breast cancer cases outcome with respect to HER2 expression which found a worse relapse-free survival among HER2-low than HER2-0 cases (Jacot et al., 2021; U.S. National Library of Medicine, 2022). We can infer that this is because in our case series patients with HER2-low/HR-negative were enriched of less differentiated tumors. Apart from this clinic-pathological characteristic, molecular features might also explain the observed outcomes. The percentage of HER2-enriched subtype has been reported to be higher in HR-negative/HER2-low than HER2-0, e.g., 13.7% versus 1.6% (Agostinetto et al., 2021). Consistent with this, the levels of *ERBB2* were higher in HER2-low than HER2-0 groups (Schettini et al., 2021). Several retrospective analyses have already been reported showing that breast cancer patients have worse outcomes as HER2 levels increase (Gilcrease et al., 2009; Rossi et al., 2012; Eggemann et al., 2015). In fact, some have put forward the hypothesis of a linear correlation between HER2 expression and tumor behavior (Eggemann et al., 2015), which, however, deserves to be demonstrated and especially evaluated separately in HR-positive and -negative disease.

Finally, an additional important hint from this study is whether we should put efforts to develop new therapeutic strategies in HER2-low breast cancer patients, since pCR retained its favorable prognostic significance independently of HER2 expression levels. The development of novel ADCs targeting HER2 has opened up a new window for the treatment of HER2-low breast cancer (Rinnerthaler et al., 2019). Results from clinical trials of the most advanced ADC, T-DXd, have shown that even a low-to-moderate expression of HER2 receptor is sufficient to trigger therapy response (Modi et al., 2020). Interestingly, the treatment efficacy seemed not to differ according to HR-status. Specifically, the objective response rate in previously treated advanced breast cancer patients was 52.6% (95%CI 47.0–58.0) in HR-positive and 50% (95%CI 33.8–66.2) in HR-negative cases. Therefore, it might be inferred that treatment with ADC could be exploitable in neoadjuvant setting to increase the rate of pCR and to ameliorate the outcome of early-stage HER2-low population irrespective of HR-status. Although there is not yet sufficiently strong clinical evidence to change the standard of care, patients with early-stage HER2-low and hormone receptor-positive breast cancer might benefit from anti-HER2 agents with or without endocrine therapy. This is the bottom line of the ongoing phase II study (NCT04553770) aiming at identifying the treatment arm with strongest signal of efficacy based on pCR between neoadjuvant deruxtecan alone or in combination with anastrozole in patients with HER2-low/HR-positive early-stage breast cancer (U.S. National Library of Medicine, 2022).

Our study has several limitations. First, this was a retrospective single-center study; thus, some imbalances between groups and referral bias might exist. However, the intra-laboratory heterogeneity of HER2 detection was somewhat avoided. Second, the HER2 status was evaluated based on the primary tumor. Re-biopsy of recurrent and metastatic lesions was not performed in the majority of patients, and the discordance of HER2 status could not be ruled out in at least 30% of cases. Third, our study did not include -omic information of HER2-low patients. Large-scale genomic analyses might shed some light on the genomic background of HER2-low patients in the near future.

## Conclusion

In this series of real-world data, HER2-low breast cancer accounted for almost a half of all cases treated with neoadjuvant therapy. Yet pCR was achieved by a minority of cases. We reported a significant association between HER2-low and reduced pCR overall. This finding was consistent with the pooled analysis of data from clinical trial (Denkert et al., 2021) and in contrast with results from retrospective studies involving few patients and showing no impact of HER2 status on response to therapy (de Moura Leite et al., 2021; Alves et al., 2022; Domergue et al., 2022). The predictive value of HER2-low appeared to be more pronounced in HR-positive than -negative cases, though the difference did not reach the statistical significance. Our exploratory analysis aiming at assessing possible survival differences according to HER2 status showed that pCR predicted favorable outcome in both HER2-low and HER2-0 cases. Notably, the prognosis of patients with HER2-low not attaining a pCR was poor, especially when primary tumor lacked HR. These results add value to the general understanding of HER2-low disease suggesting that HR is a determinant of the underlying biology of HER2-low tumors, and HR-positive/HER2-low and HR-negative/HER2-low subgroups might be identified as distinct biological entities within the HER2-negative population. Due to the retrospective nature of this study, whether we can further distinguished additional distinct subtypes among HER2-negative cases remains unknown. Further investigations aiming to characterize the more detailed molecular landscape and to understand the natural history of HER2-low population are warranted. Such studies will be instrumental for the development of ADCs in early-stage breast cancer treatment in the next future.

## Data Availability

The raw data supporting the conclusions of this article will be made available by the authors upon reasonable request
