# Supplementary material for: Moving HER2-low breast cancer predictive and prognostic data from clinical trials into the real world
Source: Front Mol Biosci. 2022 Sep 26;9:996434. doi: 10.3389/fmolb.2022.996434 (PMC9549400; doi:10.3389/fmolb.2022.996434)
Supplement: Supplementary file 1 [file Table1.DOCX]

**Supplementary Table 1.** Baseline characteristics of hormone receptor-positive breast cancer patients according to HER2-0 and HER2-low

|  | **HER2-0**  (N=47) | **HER2-low**  (N=272) | p-value |
| --- | --- | --- | --- |
| **Age** |  |  | 0.0860 |
| Mean (SD) | 54.6 (12.4) | 51.5 (11.5) |  |
| Median [Q1, Q3] | 54.1 [45.5, 62.4] | 49.5 [43.6, 59.3] |  |
|  |  |  | 0.0400 |
| <50 | 17 (36.2%) | 143 (52.9%) |  |
| >=50 | 30 (63.8%) | 127 (47.1%) |  |
| **Body mass index** |  |  | 0.5839 |
| Mean (SD) | 25.4 (5.25) | 24.6 (4.44) |  |
| Median [Q1, Q3] | 24.1 [21.3, 28.2] | 24.1 [21.3, 27.5] |  |
| missing | 6 (12.8%) | 26 (9.6%) |  |
| **Neoadjuvant chemotherapy** |  |  | 0.7505 |
| Anthracycline and taxane-based | 45 (95.7%) | 253 (93.0%) |  |
| Other | 2 (4.3%) | 19 (7.0%) |  |
| **Surgery type** |  |  | 0.2300 |
| Mastectomy | 29 (61.7%) | 193 (71.0%) |  |
| Breast conservative surgery | 18 (38.3%) | 79 (29.0%) |  |
| **Axillary dissetion** |  |  | 0.3239 |
| No | 20 (42.6%) | 94 (34.6%) |  |
| Yes | 27 (57.4%) | 178 (65.4%) |  |
| **Clinical tumor size** |  |  | 0.8345 |
| 2-5 cm | 34 (72.3%) | 185 (68.0%) |  |
| > 5 cm | 13 (27.7%) | 84 (30.9%) |  |
| missing | 0 (0%) | 3 (1.1%) |  |
| **Clinical nodal status** |  |  | 0.7868 |
| N0 | 10 (21.3%) | 71 (26.1%) |  |
| N1 | 33 (70.2%) | 181 (66.5%) |  |
| N2- 3 | 3 (6.4%) | 15 (5.5%) |  |
| missing | 1 (2.1%) | 5 (1.8%) |  |
| **Stage** |  |  | 0.8656 |
| I or II | 32 (68.1%) | 181 (66.5%) |  |
| III | 14 (29.8%) | 86 (31.6%) |  |
| missing | 1 (2.1%) | 5 (1.8%) |  |
| **Grading** |  |  | 0.4961 |
| I | 1 (2.1%) | 14 (5.1%) |  |
| II | 21 (44.7%) | 143 (52.6%) |  |
| III | 17 (36.2%) | 83 (30.5%) |  |
| missing | 8 (17.0%) | 32 (11.8%) |  |
| **Estrogen and Progesterone receptor status** |  |  | 0.2979 |
| At least one positive | 11 (23.4%) | 45 (16.5%) |  |
| Both positive | 36 (76.6%) | 227 (83.5%) |  |
| **Estrogen category** |  |  | 0.7929 |
| Negative | 2 (4.3%) | 6 (2.2%) |  |
| Low | 3 (6.4%) | 20 (7.4%) |  |
| Intermidiete | 2 (4.3%) | 13 (4.8%) |  |
| High | 40 (85.1%) | 233 (85.7%) |  |
| **Progesterone category** |  |  | 0.6673 |
| Negative | 9 (19.1%) | 39 (14.3%) |  |
| Low | 12 (25.5%) | 60 (22.1%) |  |
| Intermidiete | 7 (14.9%) | 41 (15.1%) |  |
| High | 19 (40.4%) | 132 (48.5%) |  |
| **Ki67** |  |  | 0.1531 |
| < 20% | 6 (12.8%) | 70 (25.7%) |  |
| ≥ 20% | 38 (80.9%) | 185 (68.0%) |  |
| missing | 3 (6.4%) | 17 (6.3%) |  |
| **Pathological complete response** |  |  | 0.1916 |
| No | 42 (89.4%) | 257 (94.5%) |  |
| Yes | 5 (10.6%) | 15 (5.5%) |  |

**Supplementary Table 2.** Baseline characteristics of hormone receptor-negative breast cancer patients according to HER2-0 and HER2-low

|  | **HER2-0**  (N=62) | **HER2-low**  (N=63) | p-value |
| --- | --- | --- | --- |
| **Age** |  |  | 0.2730 |
| Mean (SD) | 49.9 (9.46) | 48.0 (11.7) |  |
| Median [Q1, Q3] | 48.5 [43.6, 57.3] | 45.8 [39.8, 57.3] |  |
|  |  |  | 0.5894 |
| <50 | 34 (54.8%) | 38 (60.3%) |  |
| >=50 | 28 (45.2%) | 25 (39.7%) |  |
| **BMI** |  |  | 0.4558 |
| Mean (SD) | 24.6 (4.92) | 25.6 (5.80) |  |
| Median [Q1, Q3] | 23.6 [21.1, 27.1] | 24.2 [21.4, 27.9] |  |
| missing | 3 (4.8%) | 8 (12.7%) |  |
| **Neoadjuvant chemotherapy** |  |  | 0.8306 |
| Anthracycline and taxane-based | 49 (79.0%) | 48 (76.2%) |  |
| Other | 13 (21.0%) | 15 (23.8%) |  |
| **Surgery type** |  |  | 0.4753 |
| Mastectomy | 31 (50.0%) | 36 (57.1%) |  |
| Breast conservative surgery | 31 (50.0%) | 27 (42.9%) |  |
| **Axillary surgery** |  |  | 0.2474 |
| No | 46 (74.2%) | 40 (63.5%) |  |
| Yes | 16 (25.8%) | 23 (36.5%) |  |
| **Clinical tumor size** |  |  | 0.4824 |
| 2-5 cm | 44 (71.0%) | 48 (76.2%) |  |
| > 5 cm | 16 (25.8%) | 15 (23.8%) |  |
| missing | 2 (3.2%) | 0 (0%) |  |
| **Clinical nodal status** |  |  | 0.7887 |
| N0 | 26 (41.9%) | 24 (38.1%) |  |
| N1 | 32 (51.6%) | 34 (54.0%) |  |
| N2- 3 | 4 (6.5%) | 2 (3.2%) |  |
| missing | 0 (0%) | 3 (4.8%) |  |
| **Stage** |  |  | 0.8342 |
| I or II | 48 (77.4%) | 46 (73.0%) |  |
| III | 14 (22.6%) | 15 (23.8%) |  |
| missing | 0 (0%) | 2 (3.2%) |  |
| **Grading** |  |  | 0.0188 |
| I | 0 (0%) | 0 (0%) |  |
| II | 7 (11.3%) | 2 (3.2%) |  |
| III | 44 (71.0%) | 57 (90.5%) |  |
| missing | 11 (17.7%) | 4 (6.3%) |  |
| **Ki67** |  |  | 0.3610 |
| < 20% | 3 (4.8%) | 2 (3.2%) |  |
| ≥ 20% | 57 (91.9%) | 61 (96.8%) |  |
| missing | 2 (3.2%) | 0 (0%) |  |
| **Pathological complete response** |  |  | 0.5874 |
| No | 35 (56.5%) | 39 (61.9%) |  |
| Yes | 27 (43.5%) | 24 (38.1%) |  |
