# Supplementary material for: Moving HER2-low breast cancer predictive and prognostic data from clinical trials into the real world
Source: Front Mol Biosci. 2022 Sep 26;9:996434. doi: 10.3389/fmolb.2022.996434 (PMC9549400; doi:10.3389/fmolb.2022.996434)
Supplement: Supplementary file 2 [file Table2.DOCX]

**Supplementary Figure 1.** KM of DFS according to HER2 and HR-status.

**a)**

**
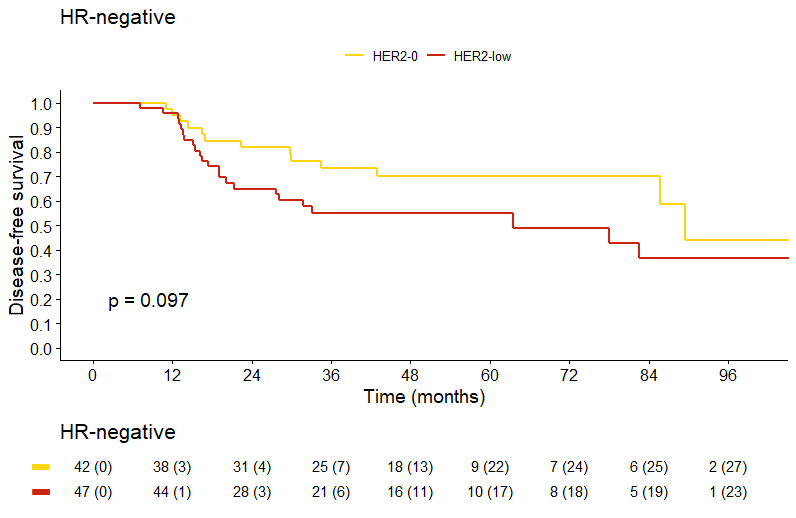
**

**b)**

**
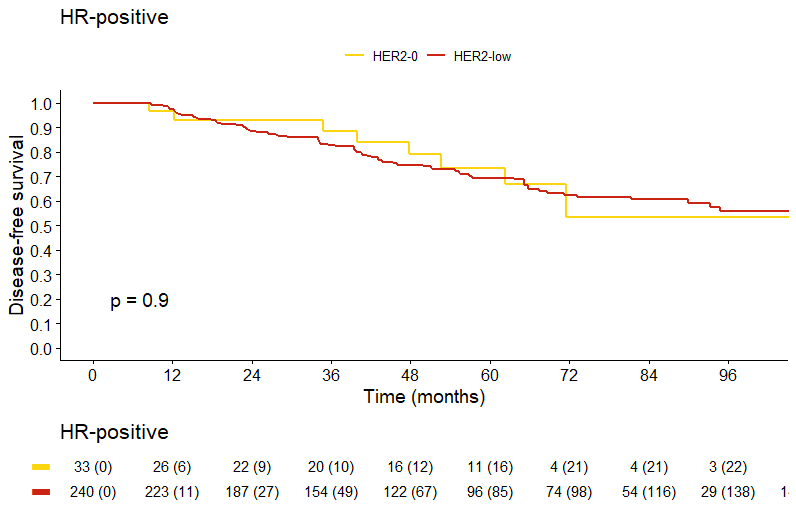
**

a) 3-year DFS estimates: HER2-0 0.73 (0.61, 0.89), HER2 -low 0.55 (0.42, 0.72); log-rank 0.097. b) 3-year DFS estimates: HER2-0 0.88 (0.77, 1.00), HER2 -low 0.83 (0.78, 0.88); log-rank 0.899

**Supplementary Figure 2.** KM of DFS according to HER2 status in HR-negative breast cancer patients achieving or not a pCR.

**a)**

**
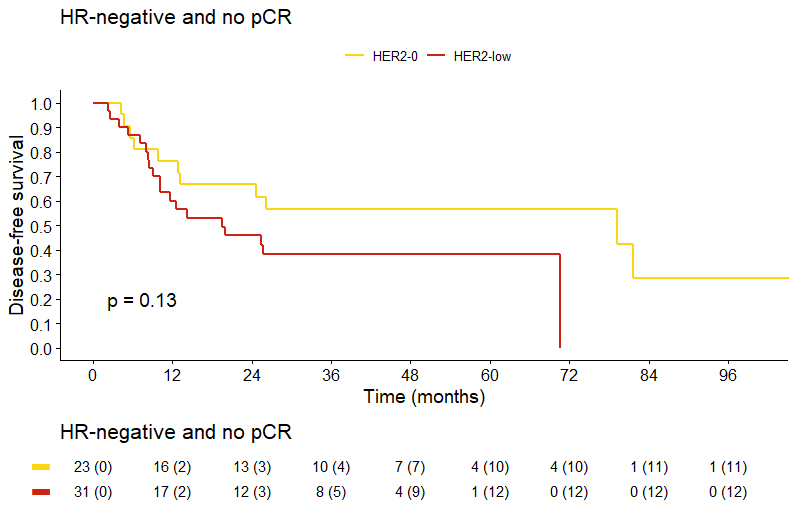
**

**b)**

**
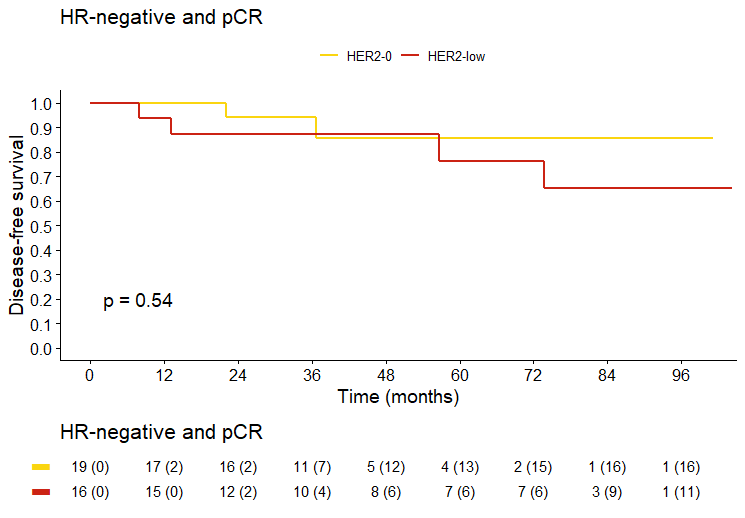
**

a) 3-year DFS estimates: HER2-0: 0.56 (0.39-0.83), HER2-low : 0.382 (0.24-0.61), Log-rank p-value=0.126; b) 3-year DFS estimates HER2-0: 0.94 (0. 84-1.00) HER2-low : 0.87 (0. 72-1.00) Log-rank p-value=0.536
